# Secondary structure and ^1^H, ^13^C, ^15^N resonance assignments of the endosomal sorting protein sorting nexin 3

**DOI:** 10.1007/s12104-015-9609-z

**Published:** 2015-04-19

**Authors:** Michael Overduin, Sandya Rajesh, Jean Gruenberg, Marc Lenoir

**Affiliations:** Henry Wellcome Building for Biomolecular NMR Spectroscopy, School of Cancer Sciences, University of Birmingham, Edgbaston, Birmingham, B15 2TT UK; Department of Biochemistry Sciences II, University of Geneva, 30, Quai Ernest-Ansermet, 1211 Geneva 4, Switzerland

**Keywords:** SNX3, PX domain, Phosphoinositide recognition, Phosphatidylinositol 3-phosphate, Endosome, NMR

## Abstract

Sorting nexin 3 (SNX3) belongs to a sub-family of sorting nexins that primarily contain a single Phox homology domain capable of binding phosphoinositides and membranes. We report the complete ^1^H, ^13^C and ^15^N resonance assignments of the full-length human SNX3 protein and identification of its secondary structure elements, revealing a canonical fold and unstructured termini.

## Biological context

The SNX3 protein is localized within the early endosome through its interaction with phosphatidylinositol 3-phosphate (PI3P) (Xu et al. 2001). It associates with the cargo-selective retromer complex VPS26-VPS29-VPS35 in a trafficking pathway, thus forming a retromer capable of transporting Wntless from the endosome to the trans-Golgi network (Harterink et al. 2011). SNX3 has also been implicated in the formation of the intra-endosomal vesicles that are found in multivesicular bodies (Pons et al. 2008) and in the maturation of the Salmonella-induced vacuole in infected cells (Braun et al. 2010). Together with the SNX10, SNX11, SNX12, SNX22, SNX23 and SNX24 proteins, SNX3 belong to a sub-family of sorting nexins that contain a single PX domain and apparently unstructured termini (Cullen and Korswagen 2012). PX domain-containing proteins are well known for their phosphatidylinositide binding activities, and are typically 120 amino acids in length. Their structural domains typically contain three antiparallel β strands followed by an unstructured loop with a conserved PXXP motif followed by three α helices. The structures, dynamics and interactions of full-length sorting nexins are less well understood.

Here, we have assigned the resonances of the wild-type human SNX3 protein to identify its secondary structure and disordered regions, revealing a classical topology with an unstructured loop at the vicinity of the PI3P binding site. The assignment of the resonances provides a basis for the structural and dynamic analysis of this full length protein and its ligand and membrane binding mechanisms.

## Methods and experimental

### Expression and purification of SNX3

A pET45b (Merck) vector including the entire *SNX3* sequence encoding residues 2–162 was expressed in *E.coli* strain BL21(DE3). Cells were grown in M9 media supplemented by ^15^NH_4_Cl and ^13^C-glucose. Expression was induced by addition of 1 mM IPTG until the OD_600_ reached 0.6. The cells were harvested by centrifugation at 6000*g* for 20 min and resuspended in 20 mM TrisHCl buffer pH 7.5, 100 mM NaCl, 1 mM DTT, 20 mM imidazole and Complete EDTA-free protease inhibitors (Roche). The cells were lysed with an Emulsiflex (17,000 psi) and the lysate was centrifuged at 75,000*g* for 45 min. The protein was bound to a HisTrap FF column (GE Healthcare) and eluted by an imidazole gradient (20–500 mM). Fractions containing SNX3 were further purified on a Superdex S75 HiLoad column connected to an ÄKTA Purifier (G.E. Healthcare) in a 20 mM sodium phosphate buffer containing 100 mM NaCl, DTT, and 1 mM NaN_3_. NMR samples were prepared with 10 % D_2_O (v/v).

### NMR spectroscopy

NMR spectra were acquired at 298 K on a Varian Inova 800 spectrometer equipped with a triple resonance ^1^H/^13^C/^15^N cryogenic probe and z-axis pulsed field gradients with a uniformly ^13^C/^15^N labeled sample of 800 μM SNX3 protein. The backbone assignment was obtained using Biopack pulse sequences (Varian) to collect HNCO, HN(CA)CO, HN(CO)CA, HNCA, CBCA(CO)NH, HNCACB, H(C)CH-TOCSY, and (H)CCH-TOCSY spectra. The ^15^N-edited NOESY-HSQC and ^13^C-edited NOESY-HSQC experiment were acquired with mixing times of 100 ms. Spectra were processed using Nmrpipe (Delaglio et al. 1995) and analyzed with the Ccpnmr suite (Vranken et al. 2005). Proton chemical shifts were directly referenced against external 4,4-dimethyl-4-silapentane-1-sulfonic acid (DSS) standard while the ^15^N and ^13^C chemical shifts were referenced indirectly from the gyromagnetic ratios (Wishart et al. 1995).

## Assignments and data deposition

**Fig. 1:**
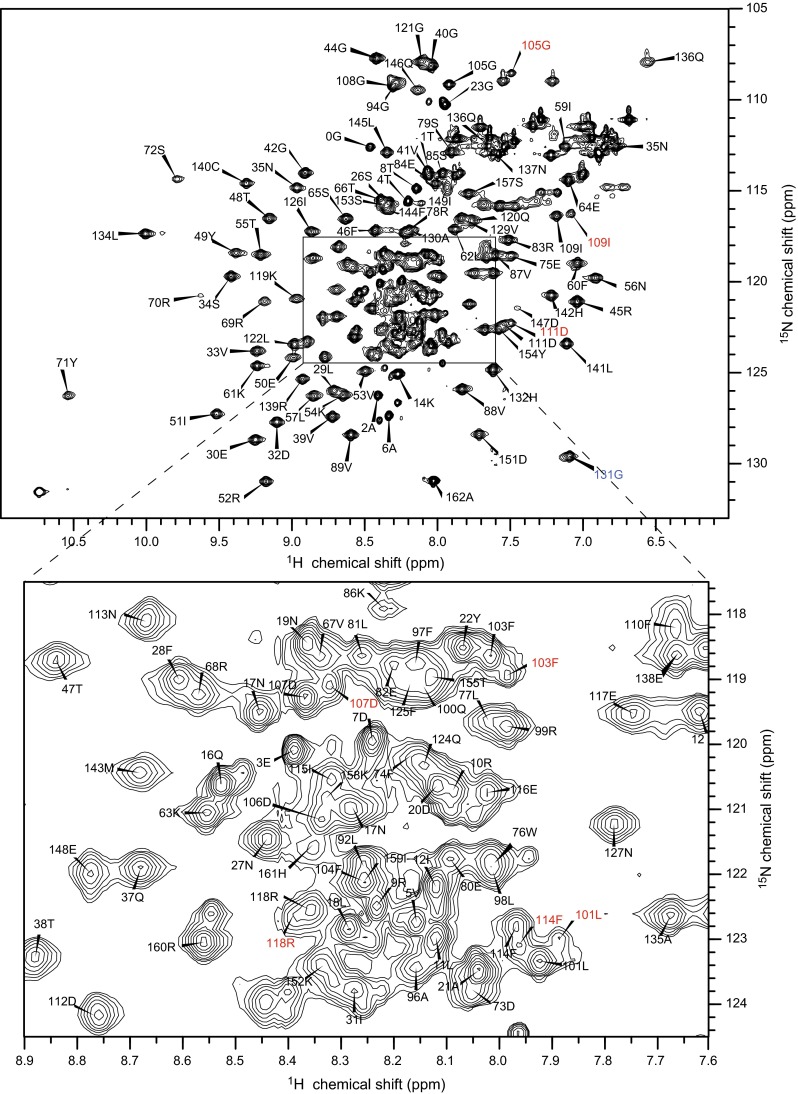
^1^H-^15^N HSQC of SNX3 (700 μM) in 20 mM sodium phosphate pH 6.5, 100 mM NaCl, 1 mM NaN_3_ and 10 % (v/v) D_2_O collected at 298 K on a Varian INOVA 800 MHz spectrometer. *Residue* *numbers* are indicated for cross peaks corresponding to backbone amides. Aliased residue peaks are indicated in *blue* and alternate conformer peaks in *red*

The ^1^H-^15^N HSQC spectrum of the full length SNX3 shows the assignment of essentially all the resonances (Fig. 1). The backbone assignment was complete except for the N-terminal His_6_ tag. Beyond the affinity tag, most of the backbone HN (99 %), non-proline N (99 %), Cα (100 %), Cβ (100 %), C′ (96 %) and Hα (100 %) resonances were assigned.

A second conformer was likely due to the isomerization of Pro102 and represented c.a. 30 % of the population based on the relative intensities of the peaks. The second set of resonances was unambiguously assigned for residues Leu101, Phe103, Gly105, Asp107, Ile109, Asp111, Phe114 and Arg118 situated within a predicted loop between α1 and α2 helices and at the start of the α2 helix while the weaker resonances of the Arg104, Asp106, Gly108, Phe110, Asp112 and Asn113 residues were overlapped or obscured.

**Fig. 2:**
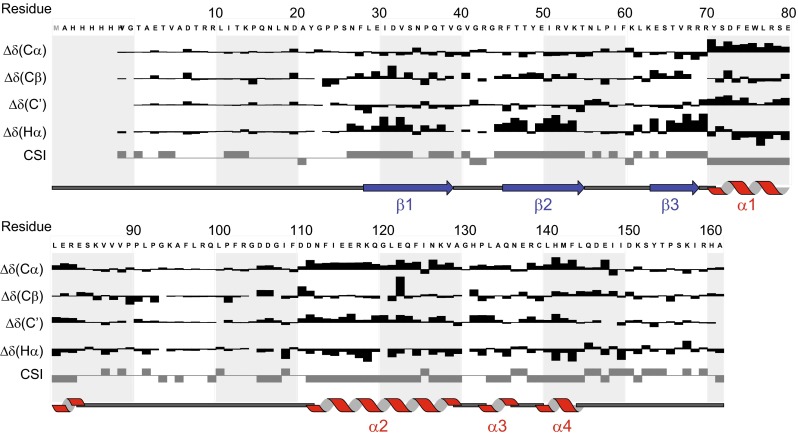
Secondary structure of SNX3 predicted by CCPNMR analysis. The *arrows* and *bars* indicate the positions of β strands and α helices, respectively

The secondary structure of SNX3 was predicted from Hα, Cα, Cβ, C′ chemical shifts using Ccpnmr analysis suite and the corresponding chemical shift index (Fig. 2). Three β-strands (29–39, 46–55, 64–69) followed by three α-helices (71–84, 112–130, 133–145) were predicted, consistent with the structural characteristics of PX domains and SNX12-PX in particular (pdb 2CSK). The third helix may contain an irregularity midway based on an interrupted CSI pattern, while both termini exhibit random coil resonances and are intrinsically disordered.

The assignments of the apo state have been deposited to the BioMagResBank with the accession number 25402.
